# Neonatal Airway Management

**DOI:** 10.3390/children11010082

**Published:** 2024-01-10

**Authors:** Joaquim M. B. Pinheiro

**Affiliations:** Division of Neonatology, Department of Pediatrics, Albany Medical College, Albany, NY 12208, USA; pinheij@amc.edu; Tel.: +1-518-262-5421

## 1. Introduction

The neonatal airway is often difficult to secure, whether the practitioner responsible for managing the airway is a neonatologist, pediatrician, anesthesiologist, another specialist or an advanced practice provider. During intubation procedures, the highest incidence of difficult airway occurs among the neonatal population [1,2]. Among intubated patients, neonates are also at the highest risk of unplanned extubation relative to any other age group [3]. While neonates are subject to the greatest difficulties during tracheal intubation, they also experience more pain and discomfort during this procedure than older patients, since they are often not adequately premedicated to minimize pain, procedure-related stress, and the possibility of procedural failure [4,5,6]. Changes in neonatal airway management practices in the last two decades, particularly with the decline in tracheal intubation in the delivery room for the suctioning of meconium or administration of surfactant, have led to a decrease in intubation opportunities for pediatric trainees. This has resulted in a marked loss of intubation experience during training, translating into a decreased level of expertise at the attending physician or consultant level [7,8,9]. At least in the United States, the advent of advanced practice providers and respiratory therapists as additional practitioners of intubation in neonatal care settings have further diluted the experience of neonatal airway management experts and of pediatric trainees. Given these trends, it is unrealistic to expect that further progress in the effectiveness and safety of neonatal airway management can be grounded on increasing the procedural expertise of individual practitioners. Rather, improvements in the proficiency of neonatal airway management must rely on increased knowledge of basic features of the airway at various stages of development and on the application of this knowledge to the design of devices optimized for neonates of various sizes. Another foundational component of improvement in this area is the development of team-based, standardized processes for airway management, for situations including two-person bag mask ventilation, tracheal intubation [10], or moving an intubated patient [11]. Optimized devices, procedural steps, and user functions will ensure the most appropriate options for safe, comfortable, and effective airway interventions in individual infants.

## 2. Overview of Published Articles

The normal growth of the developing trachea, as well as structural abnormalities of the neonatal airways, are fundamental anatomical features that must be understood to guide practice. Two articles in this Special Issue of *Children* address clinically relevant airway anatomy. Srikanthan et al., in the first contribution, review neonatal airway anomalies and associated dysfunctions, which, alone or in association with postnatal injuries, can severely impair the conducting function of the airways at birth or during the perinatal period. The authors discuss the origin, clinical manifestations, evaluation, and management of airway anomalies that may be encountered by obstetricians, pediatricians, or neonatologists caring for newly born infants; some of these anomalies require advanced airway interventions which necessitate the involvement of pediatric anesthesiologists and otolaryngologists. From this article, clinicians in all these disciplines will derive useful information on diagnostic approaches and management principles for a wide range of neonatal airway abnormalities. 

The manuscript by Cerone et al., this Special Issue’s second contribution, addresses a critical deficiency in our knowledge of the neonatal airway: understanding developmental changes in the length of the neonatal trachea, from periviable gestations to full-term newborns. This knowledge is fundamental to informing design standards of endotracheal tubes (ETTs) intended for use in newborns, as well as to the proactive design of procedures that promote a more precise—and thus safer and more efficient—insertion and positioning of the ETT. Using software integrated with digital X-ray systems, the authors obtained in vivo measurements from chest x-rays of neonates between 22 and 42 weeks of gestation. The results imply that glottic depth markings on currently available neonatal ETTs tend to promote the insertion of the tube deeper in the trachea than normally intended, particularly for newborns of extremely low birthweights. These findings should also be considered when standards for neonatal ETT design and manufacturing, including device markings and instructions that will enhance the usability of these devices, are revised by the ISO committees [12].

The paper by Pinheiro and colleagues, this Special Issue’s third contribution, provides evidence on the need for improved precision in the intratracheal placement of the endotracheal tube, focusing on the endobronchial positioning of the ETT during intubation, which remains a threat to patient safety despite successful tracheal intubation. This undesirable “plastic overdose” is a useful intermediate outcome measure of the intubation process, which may be associated with immediate morbidities related to inadequate ventilation and/or trauma, as well as delayed adverse sequelae. The authors report on the methods and results of a long-term quality improvement project involving more than 5000 consecutive intubations. Although the rates of deeply positioned ETTs declined in the early phases of the project, they were not sustained below the team’s target. The authors identify multiple factors contributing to endobronchial intubations, and suggest countermeasures aimed at improving intubation safety to be applied at all stages of the procedure: before, during, and immediately after tube insertion. The simple specification of the expected ETT depth among team members, before the start of intubation, is possibly the most effective intervention to avoid endobronchial intubations. 

One of the consequences of deeply positioned ETTs is that they need to be repositioned to a proper depth. During this repositioning, ETTs may become dislodged. Also, because the estimation of depth adjustments is often imprecise, repositioning may result in a shallow location of the ETT tip high in the trachea, thus increasing the risk of subsequent dislodgment during routine care or patient movement. These mechanisms are among the many avoidable causes of unplanned extubation in neonates—the subject of the next article.

The manuscript by Nelson et al. (the fourth contribution of this collection) addresses the difficulties in maintaining a secure airway in neonates, who are at a much higher risk of unplanned extubation (UE) than more mature patients [3]. The authors report an improvement collaborative among four large regional NICUs in which the centers first agreed on an unequivocal operational definition of UE, namely, “any extubation that was not pre-planned for that time, or not carried out electively”, to account for all unplanned or untimely displacements of the ETT from the trachea, regardless of whether this occurred through the actions of patients, caregivers, equipment, or other causes [13]. Previously, there had been disparate interpretations of this definition among centers. The collaborative succeeded in decreasing the rates of UEs, primarily by diminishing the frequency of ETTs removed by staff, suggesting that some of the emergency extubations were avoidable. However, the rates of dislodgment of ETTs from the trachea remained higher than desired, due to a combination of patient actions and failure of securing devices and caregivers to maintain the ETT in a stable position. Despite collaborative learning regarding methods to keep ETTs stable, the lack of progress in this area indicates that much more work needs to be carried out on approaches to safely securing ETTs in neonates.

The study by Abu Leyah and colleagues, the fifth contribution to this Special Issue, explores laryngeal mask-type devices as an alternative approach to avoid both laryngoscopy and tracheal intubation in newborns who need surfactant therapy for respiratory distress syndrome. The authors showed that surfactant administration through laryngeal or supraglottic airways (SALSA) can be adopted and implemented rapidly, even in low- to middle-income country settings. Undoubtedly, this approach could be adopted easily in NICUs with greater technical resources. However, published examples of routine SALSA implementation are still scarce [14]. This is likely explainable by the very limited experience of neonatologists with laryngeal mask use [15,16,17] for its primary indication, delivery room resuscitation [10]. The unavailability of laryngeal mask/supraglottic airway devices appropriately sized for extremely low birthweight neonates restricts the applicability of SALSA to neonates weighing more than 800 g, even under research conditions [18]; guidelines from other experienced groups restrict their use of size 1 laryngeal mask devices to neonates who weigh more than 1250 g [19]. Since it is possible to manufacture a laryngeal mask device for use in rats [20], there should be few technical barriers to developing such devices for the benefit of human newborns. It is likely that the perceived low demand for these devices is a limiting factor for their development for use in small, preterm, human newborns. The work of Abu Leyah et al. should encourage clinicians who care for neonates to adopt SALSA in their practice settings, to acquire basic comfort with laryngeal mask device use, and should rapidly increase the demand for these devices, which should, in turn, augment manufacturers’ interest in developing a variety of these simple tools optimized for use in neonates.

## 3. Conclusions

As in most aspects of medical care, neonates needing critical airway management for life support are “therapeutic orphans” since they constitute a relatively small proportion of the market share for airway-related devices. An additional impediment is the greater technical difficulty in developing monitoring and support devices that function reliably in small patients, whose sizes nevertheless vary over more than a tenfold range.

I believe that the compilation of manuscripts in this Special Issue will stimulate clinicians and researchers to focus on the special needs of neonates, for whom airway care is often critical. These contributions identify many gaps, stretching from fundamental knowledge of anatomical dimensions of the developing airway to optimally designed artificial airway devices and supporting equipment, including the safe and effective use of these tools by clinical care teams. Some ideas contained in these articles can be immediately implemented, while others provide a blueprint for future research.
